# Expectation dynamically modulates the representational time course of objects and locations

**DOI:** 10.1162/IMAG.a.999

**Published:** 2025-11-10

**Authors:** Margaret Jane Moore, Amanda K. Robinson, Jason B. Mattingley

**Affiliations:** Queensland Brain Institute, University of Queensland, St. Lucia, Queensland, Australia; School of Psychology, University of Queensland, Brisbane, Australia; Canadian Institute for Advanced Research, Toronto, Canada

**Keywords:** predictive coding, EEG, multivariate pattern analysis, decoding, expectation

## Abstract

Past work has demonstrated that predictive information modulates how the brain responds to visual stimuli, but it is not yet clear how the brain integrates different types of predictive information to facilitate efficient perception. Here, we aim to explore how expectations about upcoming stimulus identities (“what” information) and upcoming stimulus locations (“where” information) modulate the directionality and occurrence of prediction effects in brain activity. Participants (n = 40) viewed real-world object images in rapid serial visual presentation (RSVP) streams which were predictable in terms of both object identity and stimulus location. Multivariate pattern analyses of electroencephalography (EEG) data were used to quantify and compare the degree of information represented in neural activity when stimuli were random (unpredictable), expected, or unexpected in terms of identity and location. Decoding accuracy for expected locations was significantly reduced relative to random locations between 160 and 238 ms post-onset. However, this effect subsequently reversed with decoding accuracy for expected locations becoming higher than accuracy for random locations between 273 and 430 ms. This temporally dynamic effect was not replicated within analyses decoding object identity. However, consistent evidence for reduced decoding of unexpected relative to random stimuli in later time windows (>250 ms) post-onset was identified across both stimulus types (e.g. objects and locations). These results are critically important when considered in the context of predictive coding research as they highlight important complexities in how predictability modulates neural responses.

## 1 Introduction

Since their inception, predictive coding theories have revolutionised the conceptual framework that has been used to interpret critical variability in the magnitude and fidelity of task-related functional neural data (e.g. fMRI, EEG) (Mumford, 1992; Rao & Ballard, 1999; Srinivasan et al., 1982). Predictive coding models assert that sensory inputs are compared with top–down predictions at multiple processing levels in the brain (Millidge et al., 2021; Summerfield & Egner, 2009). Events that align with predicted properties are thought to be processed more efficiently, while events that violate predictions generate prediction errors that are used to update stored models of the world (Millidge et al., 2021; Summerfield & Egner, 2009). Past work has suggested that predictive information modulates how the brain responds to visual stimuli (Kaposvari et al., 2018; Moore et al., 2024; Smout et al., 2019; Tang et al., 2023), but it is not yet clear how neural circuits integrate different types of predictive information to facilitate efficient visual perception. Here, we used electroencephalography (EEG) to examine how predictions about upcoming stimulus identities (“what” information) and upcoming stimulus locations (“where” information) affect patterns of brain activity.

Past research has demonstrated that predictive information about what stimuli are expected and where these stimuli are expected to appear is represented in the brain, but it is not yet clear whether these two prediction types have analogous modulatory effects on brain activity (Ekman et al., 2017; Feuerriegel et al., 2021; Hogendoorn & Burkitt, 2018; Tang et al., 2018). In terms of “what” predictions, several previous studies have evaluated how expected and unexpected stimulus identities differ in the magnitude and fidelity of evoked patterns of brain activity. A body of experimental work has suggested that expected stimuli elicit reduced neural responses relative to stimuli that are unexpected (Kaposvari et al., 2018; Richter et al., 2018). However, prediction effects have also been found to modulate the degree of stimulus-specific information represented in corresponding brain signals. For example, recent work employing inverted encoding analyses has shown that orientation information that violates expectations yields better representational fidelity than orientations that fulfil expectations (Smout et al., 2019; Tang et al., 2018, 2023). Several studies employing more complex visual stimuli (e.g., object images) have found no difference in representational fidelity between expected and random (i.e., unpredictable) object stimuli (den Ouden et al., 2023, 2024; Moore et al., 2024). However, in recent work we found that unexpected objects are represented with reduced fidelity relative to random objects (Moore et al., 2024). Considered cumulatively, these previous studies suggest that predictions relating to the identity of expected stimuli modulate the representational fidelity of this information in corresponding patterns of brain activity.

In contrast, previous work has suggested that predictions about the likely spatial location of future stimuli seem to modulate *when* stimulus-specific information is represented in the brain (Blom et al., 2020; Ekman et al., 2017; Hogendoorn & Burkitt, 2018). For example, Hogendoorn and Burkitt (2018) found that information about the upcoming location of a visual stimulus is represented in EEG activity approximately 16 ms earlier for expected stimuli relative to unpredictable stimuli. Similarly, Ekman et al. (2017) measured brain activity using functional magnetic resonance imaging (fMRI) while participants viewed stimuli that were presented over a predictable sequence of locations. Once participants became familiar with the sequence, presentation of a stimulus at the first location alone generated a time-compressed sequence of activations in the primary visual cortex (V1) which resembled the pattern present when the full stimulus sequence was presented. It is unclear, however, whether such location-based prediction effects co-occur with, or are analogous to, the “what”-based prediction effects outlined above. Previous studies have found that BOLD responses elicited by stimuli at unexpected locations are larger than those elicited by stimuli at expected locations (Alink et al., 2010; Shen et al., 2007), but evidence from EEG studies has been less consistent. Some previous EEG work has documented larger ERP amplitudes for stimuli at expected relative to random locations (Doherty et al., 2005), whereas other work has found no difference in ERP amplitude for stimuli at expected versus unpredictable locations (Pollux et al., 2011; Pollux & Guo, 2009). Whyte et al. (2020) investigated multivariate EEG representations of locations and objects under varying expectation conditions and found that decoding accuracy was not modulated by expectation condition.

In the current study, we aimed to evaluate and compare how predictions pertaining to stimulus identity and stimulus location modulate the representational fidelity of stimulus-specific information. Specifically, we used EEG and multivariate decoding to quantify differences in neural representations of familiar, real-world objects when either their identity or their spatial location was expected, unexpected, or random. Univariate analyses have frequently been used to characterise differences in response magnitudes across prediction conditions (e.g., Amado & Kovács, 2016; Kaposvari et al., 2018), but these analyses cannot reveal stimulus-specific information such as object identity. In addition, predictive coding does not make clear predictions about the magnitude of univariate activity in macroscopic brain regions in predictable versus unpredictable conditions (Millidge et al., 2021). It is, therefore, important to characterise representational fidelity because any effects of predictability could modulate the precision of stimulus-specific neural representations without yielding a group-level difference in signal amplitude (Moore et al., 2024; Summerfield & Egner, 2009). Multivariate decoding approaches are data driven and aim to characterise complex activity patterns which capture categorical relationships between stimuli and brain responses independently of any user-defined models (Carlson et al., 2020; Oosterhof et al., 2016; Robinson et al., 2023). Previous work has used this technique to investigate how prediction effects modulate multivariate representations of visual stimuli (den Ouden et al., 2023; Moore et al., 2024; Whyte et al., 2020).

We recorded participants’ brain activity using EEG as they viewed stimulus sequences of familiar, real-world object images. These object-image sequences were statistically structured using probabilistic cueing, and participants learned predictive relationships between pairs of stimuli through exposure to this structure. Multivariate pattern analysis was used to quantify the extent to which the neural representations of objects were modulated by whether they were expected, unexpected, or unpredictable (i.e., random) in terms of their identity (“what” information) or their location (“where” information). To anticipate, we observed robust and temporally dynamic prediction effects for both what and where information, but the representational fidelity of neural activity was both stronger and more prolonged for object location information than for object identity. We offer suggestions as to why our task might have yielded such distinct patterns of prediction-related modulation of “what” and “where” information.

## 2 Methods

### 2.1 Participants

Forty-two neurotypical adult participants were recruited from The University of Queensland and were compensated for their time at a rate of AUD 20 per hour. Two participants were excluded from the final dataset (one for poor quality eye-tracking data and one for chance performance on the behavioural task), resulting in a final sample of 40 participants (29 female, 3 left-handed, average age = 24 years, range = 18–38 years). All included participants reported normal or corrected-to-normal vision and provided informed consent in writing. The study procedure was approved by The University of Queensland Human Research Ethics committee (HREA 2016001247).

### 2.2 Paradigm

**Fig. 1.**
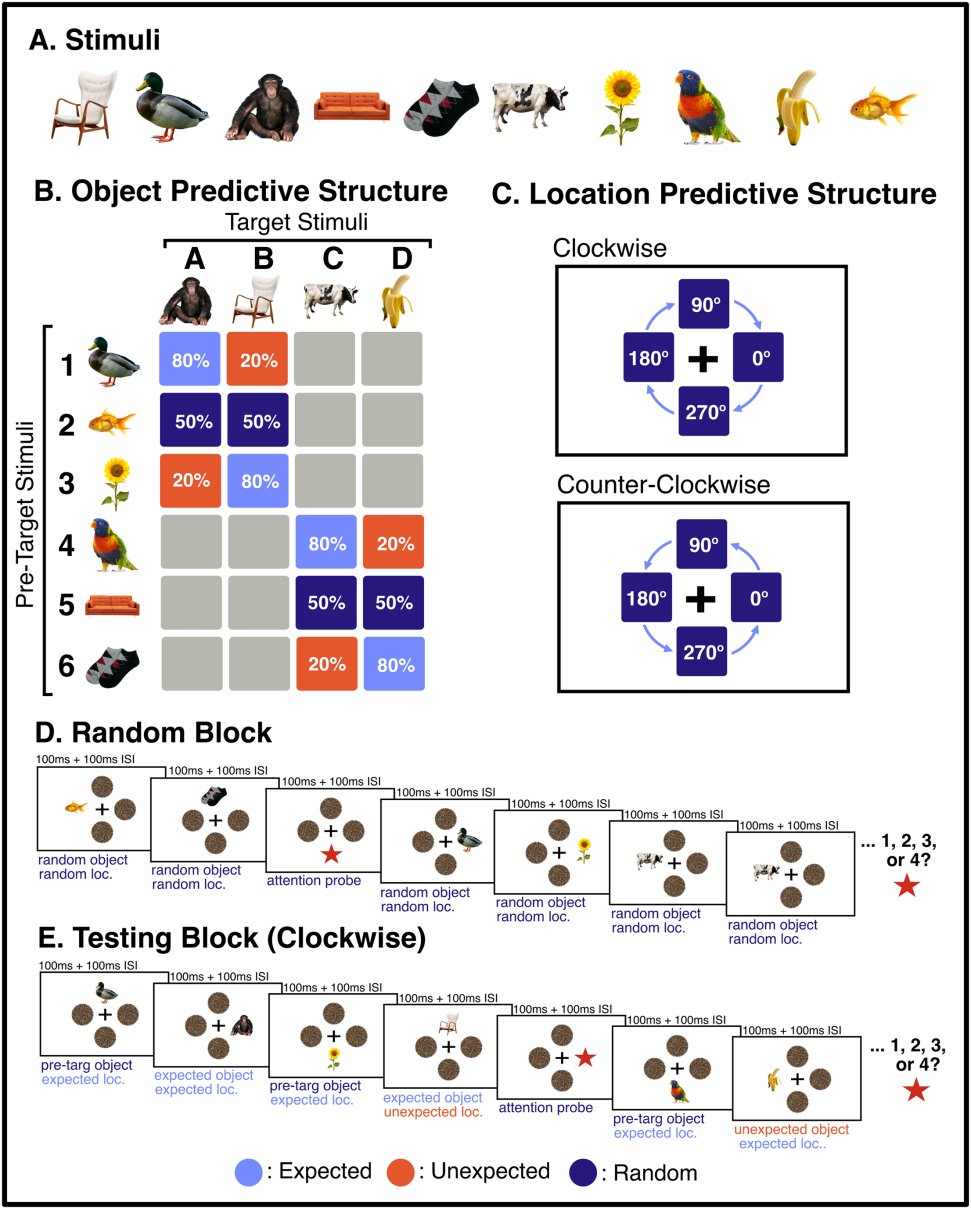
Illustration of the stimuli used in the experiment, expectation conditions, and example sequences from the random and testing blocks. (A) The 10 object stimuli used in the experiment. (B) Schematic of the statistical relationships between stimuli in the RSVP streams. Percentages denote the probability that each pre-target stimulus was followed by each possible target stimulus. There were four different relational sets (see Supplementary Materials for all four sets). (C) Location-level predictive structure. Each RSVP sequence commenced at a random location and continued either clockwise or counterclockwise (counterbalanced across blocks). Unexpected events occurred when a stimulus appeared at a location that was not the next sequential location. (D) Example displays in Random Block. (E) Example displays in Testing Block. Each stimulus was on screen for 100 ms (followed by a 100 ms ISI). All object stimuli were 3.25° in visual angle and were centred 2.75° from central fixation. Participants were instructed to maintain fixation and to silently count the number of attention probes (red stars, 1–4) within each RSVP stream.

We aimed to characterise neural representations of familiar objects presented in expected or unexpected positions within rapid serial visual presentation (RSVP) sequences displayed at four possible locations around fixation. Visual stimuli consisted of 10 familiar objects obtained from ww.pngimg.com (see Fig. 1A). These images have been demonstrated to yield reliable object-identity decoding in similar RSVP paradigms using EEG (Grootswagers et al., 2019, 2021). Perceptual expectations were induced by introducing a statistical structure to the order in which locations and objects were presented. For object-level predictions, each stimulus was classed as either a “pre-target” object or a “target” object (see Fig. 1). Each pre-target preceded one of two possible target stimuli with one of three probabilities: 20%, 50%, or 80%. For location-based predictions, objects were presented sequentially at one of four locations, in either a clockwise or counterclockwise direction (counterbalanced across RSVP streams) (Fig. 1). A given stimulus was “expected” when it appeared at the next location in the direction of the sequence, and “unexpected” when it appeared at any of the other locations.

All RSVP streams were presented at 5 Hz (100 ms stimulus exposure, 100 ms inter-stimulus interval (ISI)). Each stimulus presentation consisted of a single object image presented at one of the four possible locations. To reduce attentional capture effects (e.g., Yantis, 1993, 1996), static visual noise masks were presented at the remaining three locations (random noise masks, 3.25° visual angle). To ensure participants monitored the objects, attentional probe stimuli (red stars) were presented one to four times in each RSVP stream (see Grootswagers et al., 2019). These attentional probes were pseudo-randomly distributed with the constraint that they could not appear within the first or last 10 images, and that a minimum of 10 images appeared between each attention probe. Probes were also never placed between a pre-target and a target stimulus. RSVP streams contained 201–204 stimulus exposures (depending on the number of attentional probes), lasting approximately 40 seconds in total. Participants were instructed to maintain central fixation while counting the number of attentional probes presented in each RSVP stream. At the end of each stream, participants were prompted to report how many attention probes they had seen using numbered keys (1–4), and were provided feedback on their accuracy. Participants viewed a total of 67 RSVP streams organised into 2 distinct block types—Random and Testing—as described in detail below.

#### 2.2.1 Random block

The purpose of the Random Block was to estimate neural representations for each object where no predictive information was present. This block included the first 13 RSVP streams, including approximately 60 presentations of each object at each location. All stimulus locations and identities were presented in a *random order* with no underlying statistical structure. These data were used to train and test the classifiers when no predictive information was present (i.e., random condition decoding). In location-based decoding analyses, classifiers were trained to distinguish between all pairwise combinations of the four possible locations. In object-based decoding analyses, classifiers were trained to distinguish between all possible combinations of the 10 object stimuli at each location separately (i.e., 4 classifiers, each considering data at one location only).

#### 2.2.2 Testing block

The purpose of the Testing Block was to generate the data needed to evaluate whether neural decoding of stimulus identity and stimulus location differed across expectation conditions (expected vs. unexpected) relative to random presentations (i.e., in which there was no predictive information about object identity or location). The Testing Block included 60 RSVP sequences (~275 exposures per stimulus, per location).

Within Testing Block sequences, the identity of presented stimuli followed a predefined statistical structure (see Fig. 1). Six stimuli were “pre-targets,” each of which predicted the identity of one of two possible subsequent “targets” with a different probability. For each pair of predicted target stimuli (e.g. A & B), pre-target 1 was followed by A in 80% of trials and B in 20% of trials. Pre-target 2 was followed by A in 50% of trials and B in 50% of trials. Pre-target stimulus 3 was followed by target A in 20% and B in 80% of trials. This structure was repeated for pre-targets 4–6, which predicted target stimuli C and D (Fig. 1B). Each stimulus set consisted of six pre-target stimuli, which predicted the occurrence of four subsequent target stimuli. The identity of each pre-target and target stimulus was randomly assigned for each participant according to one of four possible relational sets (see Supplementary Materials). Attentional probe sequence positions were selected to ensure they did not interrupt this identity-level predictive structure.

For the location-based predictive structure, each Testing Block RSVP stream followed either a clockwise or counter-clockwise order of presentation (counterbalanced across sequences). Each RSVP stream began at 1 of the 4 display locations (randomly selected) and followed the expected-location sequence, with the exception of 10 unexpected-location events within each RSVP stream. These unexpected-location events occurred when a stimulus was presented in a location which was not the next sequential clockwise/counter-clockwise position. These unexpected-location events were pseudo-randomly distributed such that they were approximately evenly spaced throughout the RSVP sequence but could not occur in the first or last 10 stimuli in a block. This constraint yielded an average rate of one unexpected-location event for every 18 expected-location events. Stimulus locations were classed as expected when they were consistent with the clockwise or counter-clockwise rotating sequences, and were classified as unexpected when they violated the sequential pattern. Following each unexpected-location event, the positions of subsequent stimuli continued in the relevant direction (clockwise/counterclockwise). The first five stimuli in each stream were not included in the analyses, to ensure that the results were not influenced by stimulus events that appeared before participants had been able to discern the relevant direction of successive stimuli. The full experiment lasted approximately 60 minutes, and participants were encouraged to take regular rest breaks between RSVP streams.

### 2.3 EEG recordings and pre-processing

Continuous EEG data were recorded using a 64-electrode BioSemi system and digitised at a sampling rate of 1000 Hz. Electrodes were arranged according to the international standard 10–10 electrode placement system (Oostenveld & Praamstra, 2001). EEGLAB (Delorme & Makeig, 2004) was used for data pre-processing. Raw EEG recordings were re-referenced to mastoid channels and were filtered using low (100 Hz) and high pass (0.1 Hz) frequency filters. Noisy electrode channels were identified using joint probability, and channels were rejected if they exceeded 5 standard deviations from the average (mean number interpolated = 1.65 electrodes, SD = 1.64, range = 0-7). Removed channels were reconstructed using spherical interpolation. Data were then down-sampled to 256 Hz, segmented into stimulus-locked epochs (time interval = -100 ms to +1000 ms from stimulus onset), and baseline corrected. All reported time points are rounded to the nearest millisecond.

Central fixation was controlled and monitored using a video-based infra-red eye tracker (EyeLink 1000 Plus; SR Research). The eye tracker was calibrated at the beginning of the experiment using a standard nine-point calibration. RSVP streams only began after gaze was maintained for 500 ms on a central fixation cross presented at the beginning of each trial. If this criterion was not met within 3 seconds of fixation presentation, the calibration procedure was repeated. All EEG epochs in which an eye-blink occurred or in which fixation was not maintained within -200 to +200 ms relative to stimulus onset were excluded from subsequent decoding analyses. No other pre-processing or data cleaning was performed.

### 2.4 Decoding analyses

EEG data were analysed using a regularised linear discriminant analysis (LDA) multivariate pattern analysis (MVPA) decoding pipeline implemented in the MATLAB package CoSMoMVPA (Grootswagers et al., 2017; Oosterhof et al., 2016). This approach quantifies the discriminability of stimuli from neural activity to estimate the degree of stimulus-relevant information carried by neural signals. All decoding analyses were performed at the participant level, but overall results were analysed at the group level. Each analysis involved pair-wise decoding of either object identity or location, with classifiers trained and tested at each time point independently. These linear discriminant analysis classifiers employ ridge regularisation (penalty term = 0.01). Pair-wise decoding evaluated the discriminability of each pair of objects. For object identity analyses, each of the six possible pairwise combinations of the four target stimuli were decoded (monkey vs. chair, chair vs. cow, cow vs. monkey, etc.). To account for differences in neural representations across display locations, all models used to decode object identity were trained and tested on stimuli presented at each location independently. The final identity-level decoding models, therefore, represented the average accuracy of four independent models trained to discriminate object identity at each of the possible locations. For location analyses, the six possible combinations of the four stimulus locations were decoded. Group-level decoding of mean accuracy was calculated by averaging classifier performance for all evaluated stimulus pairs across all participants at each time point independently. This yielded a chance level of 50%.

Each decoding analysis involved training classifiers on object identity or location from the Random Block, which contained no predictive statistical structure, and testing this classifier model on trials from expected and unexpected stimuli in the Testing Block. This was done by randomly selecting 80% of relevant trial epochs to train classifiers to distinguish between each object identity or location. This process was repeated five times, each time leaving out a different 20% of relevant training epochs. These models formed the basis for comparing object identity and location representations across all conditions. To quantify representations of randomly presented object identity and location, models were tested on the left-out 20% of Random Block epochs. This cross-validation process was repeated five times for each classifier, and the mean classification accuracy at each considered time point was calculated.

In cases where classifiers were trained and tested on stimuli in different prediction conditions (e.g. trained on random from the Random Block, tested on expected or unexpected from the Testing Block), identical epochs and classification schemes were used to train the models, but these classifiers were tested on data from another condition. This approach means that, for each decoding analysis, all models were trained on identical data, thereby permitting direct comparison of neural discriminability of identical stimuli presented under random and structure (predictive) conditions. The number of trials included in each decoding model is reported in Supplementary Table 1. Due to the pairwise classification scheme, chance decoding accuracy was 50%.

### 2.5 Statistical inference

For each analysis, statistical testing was conducted to evaluate whether stimulus-specific information was present in the recorded neural data. Importantly, all between-model comparisons evaluated differences between random stimuli from the Random Block and expected/unexpected stimuli from the Testing Block. This approach avoids confounds that could arise from any putative attentional, pupillary, or domain-general differences between expected and unexpected stimuli (Feuerriegel et al., 2021). Bayes factors (BF) were computed using the BayesFactor R package to quantify the strength of evidence that decoding accuracy was above chance, and to quantify any difference in decoding accuracy across conditions (Dienes, 2011; Rouder et al., 2009; Teichmann, 2022). These tests employed alternative hypotheses with JZS priors with a default scale factor of 0.707 and a chance-level null hypothesis prior. Bayes factors were calculated to represent the probability of the observed data occurring under the alternative hypothesis relative to the null hypothesis. Bayes factors of >10 were classed as strong evidence supporting the alternative hypothesis, and BFs <1/3 were interpreted as strong evidence favouring the null hypothesis (Jarosz & Wiley, 2014; Rouder et al., 2009). Bayes factors between 3 and 10 were interpreted as representing moderate evidence in support of the alternative hypothesis.

In addition to the BF analyses, we also performed frequentist cluster-based permutation tests corrected for multiple comparisons. For these corrections, t-tests were conducted at each time point (one-tailed for comparisons vs. chance, two-tailed for cross-model comparisons). p-values <0.01 were included in clusters, and permutations (n =10,000) were conducted to quantify the probability that the summed t-score of each defined cluster occurred due to chance. All clusters yielding cluster-level p-values of <0.05 were considered significant.

## 3 Results

### 3.1 Behavioural results

All included participants performed above chance accuracy (1 to 4 targets; 25%) on the attention-probe task, with an average accuracy of 82.9% (SD = 11.1%, range = 55.2%–100%). Participants were also able to reliably maintain fixation, with an average of 83.6% of epochs (SD = 11.0%, 53.4%–99.8%) surviving eye-blink/movement-related exclusion criteria.

### 3.2 Object-level versus location-based prediction effects

**Fig. 2.**
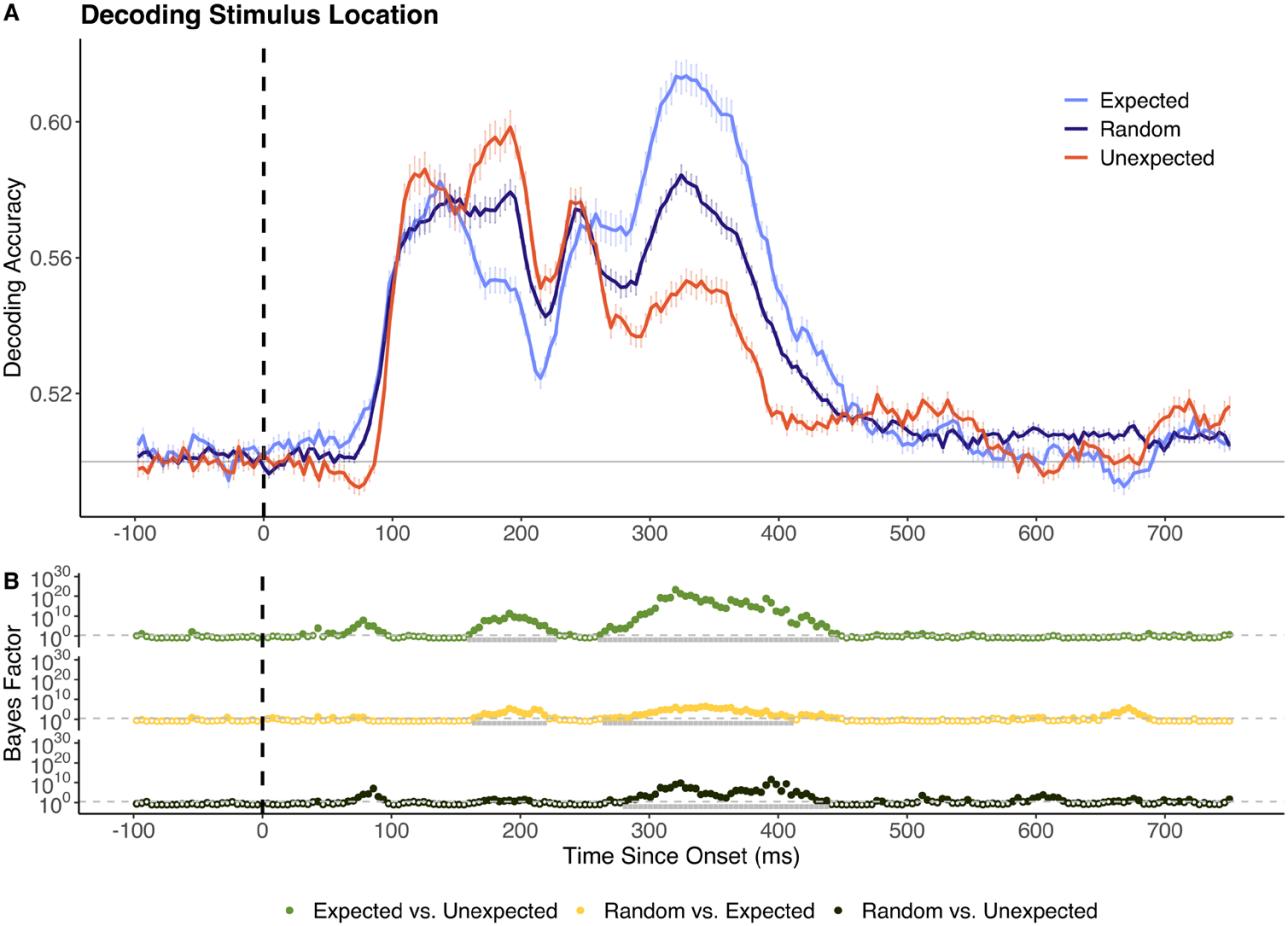
Decoding accuracy for stimuli at random, expected, and unexpected locations. (A) Sample mean decoding accuracy across time (x-axis in ms). Chance performance (50%) is denoted in grey and stimulus onset is denoted by the dotted vertical line. (B) Bayes factor plots for between-model comparisons. The dotted grey line denotes the boundary for moderate evidence (BF = 3). Tests yielding BF > 3 are represented by filled dots. Time windows in which differences remained significant following cluster-based permutation corrections are highlighted in grey. All reported time points are rounded to the nearest millisecond.

Decoding analyses were performed to determine whether prediction condition modulated the representational fidelity of object-level and/or location-based visual information. To do this, classifiers were trained to distinguish random objects/locations, then tested on data from objects/locations in expected and unexpected sequence positions. As shown in Figure 2, between 160 and 238 ms after stimulus onset decoding accuracy for expected locations was significantly *lower* than for random locations. Thereafter, from 273 to 430 ms after stimulus onset, decoding accuracy was significantly *higher* for expected locations than for random locations. Temporally dynamic prediction effects were also present for stimuli at unexpected locations. Unexpected-location events yielded significantly higher decoding accuracy than random locations between 160 and 226 ms post-onset. In a later window (289–422 ms post-onset), this relationship reversed, with unexpected location events now yielding significantly lower decoding accuracy than random stimuli. These temporally dynamic prediction effects were broadly comparable for the best and worst performing participants in the attention probe task (highest versus lowest quartile, n = 7 in each group; see Supplementary Fig. 1).

Next, we examined decoding of object identity. In the random condition, object decoding accuracy was significantly above chance (BF > 10) between 109 and 398 ms following stimulus onset. Notably, peak decoding accuracy was comparatively low for random objects (max accuracy = 52.9% at 246 ms). Decoding accuracy for unexpected objects was significantly lower than decoding accuracy for random stimuli between 277 and 289 ms post-onset (permutation-corrected cluster p-value = 0.01, mean BF = 19.36). No other significant differences were identified in decoding accuracy between expected, unexpected, and random stimuli.

### 3.3 Prediction effects are present in some, but not all decoding analyses

Analyses were conducted to evaluate whether the documented location-based prediction effects were consistent across different analysis approaches. First, we aimed to determine whether differences in location condition (i.e., expected versus unexpected, irrespective of stimulus identity) could be accurately decoded. Classifiers were trained to distinguish between stimuli appearing in expected versus unexpected locations within the Testing Block. Critically, these classifiers had no information about which specific location the stimulus had appeared in, only whether the location was expected or unexpected. Above-chance decoding from such an analysis would indicate that stimulus expectancy is, to some extent, represented in a way which is not stimulus specific, an outcome which could arise from overall differences in signal amplitude or timing between expected and unexpected conditions.

**Fig. 3.**
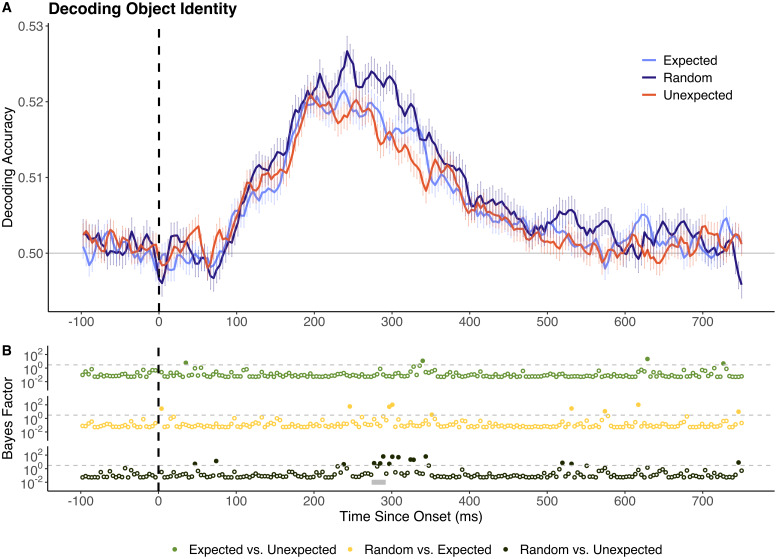
Decoding accuracy for random, expected, and unexpected objects. (A) Sample mean decoding accuracy across time (x-axis in ms). Chance performance (50%) is denoted in grey and stimulus onset is denoted by the dotted vertical line. (B) Bayes factor plots for between-model comparisons. The dotted grey line denotes the boundary for moderate evidence (BF = 3). Tests yielding BF > 3 are represented by filled dots. Time windows in which differences remain significant following cluster-based permutation corrections are highlighted in grey. All reported time points are rounded to the nearest millisecond.

This analysis yielded no reliable above-chance decoding, replicating the null result reported by den Ouden et al. (2023) (Fig. 3). Specifically, peak decoding accuracy (51.0%) occurred at 187 ms post-onset, yielding a BF of 2.67. The only time point yielding a BF > 10 occurred at 8 ms post-onset (BF = 10.76), which is well before the expected onset of decoding for visually presented stimuli information (80 ms, Carlson et al., 2013). No above-chance decoding of location condition survived cluster-based corrections for multiple comparisons.

**Fig. 4.**
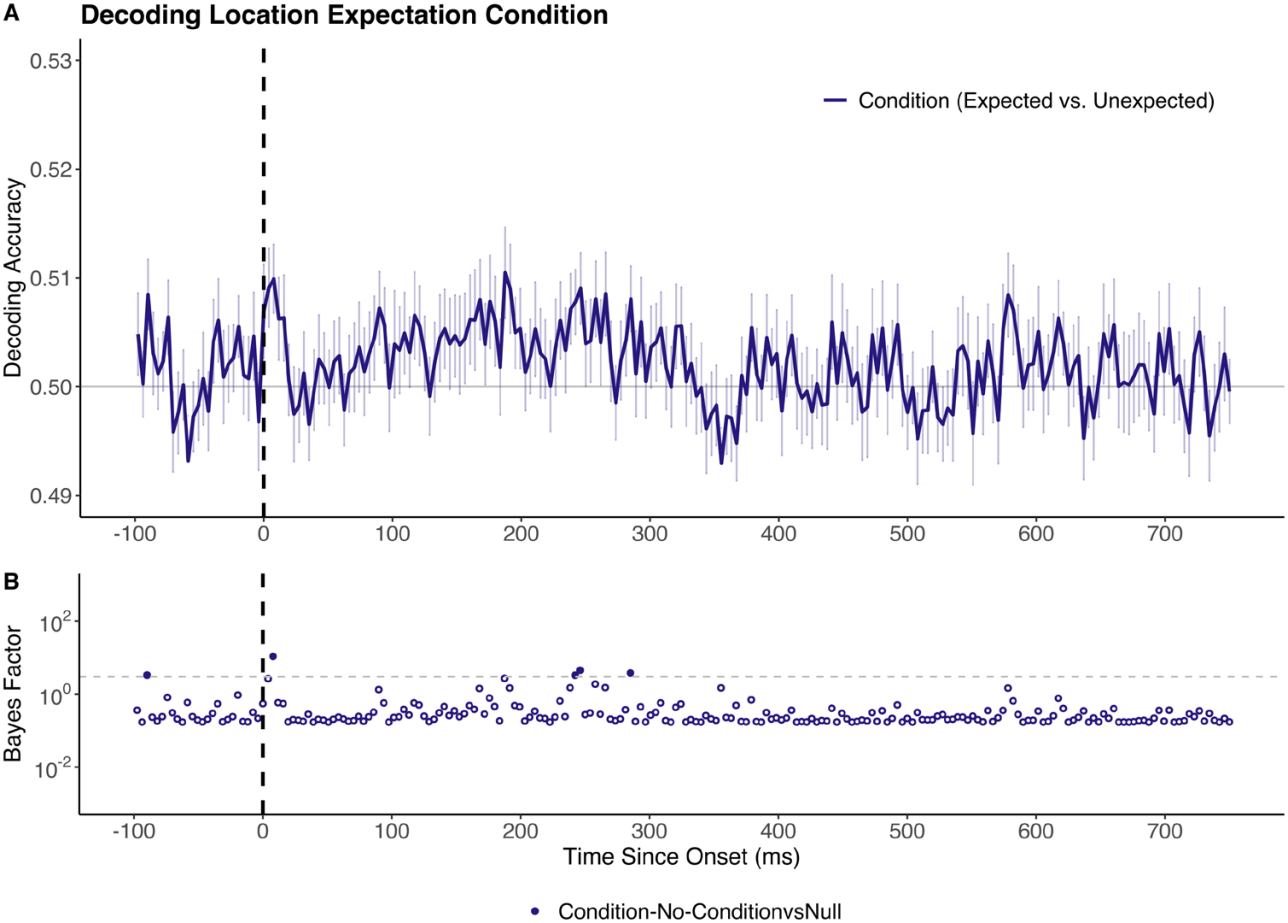
Decoding accuracy for classifiers trained to distinguish expected versus unexpected locations. (A) Sample mean decoding accuracy across time (x-axis in ms). Chance performance (50%) is denoted in grey and stimulus onset is denoted by the dotted vertical line. No above-chance decoding was identified in this analysis. (B) Bayes factor plots for between-model comparisons. The dotted grey line denotes the boundary for moderate evidence (BF = 3). Tests yielding BF > 3 are represented by filled dots. Time windows in which differences remain significant following cluster-based permutation corrections are highlighted in grey. All reported time points are rounded to the nearest millisecond.

Next, we aimed to determine whether the object-location prediction effects shown in Figure 2 were consistent when controlling for the location of the previous stimulus. These analyses were conducted to evaluate whether differences in decoding accuracy between expected and unexpected locations may have been due to representations of preceding stimuli remaining active. Recall that expected stimuli were always preceded by an immediately adjacent stimulus (90° in either direction; see Fig. 1C), whereas unexpected stimuli were preceded by more distant stimuli (180° or 270° differences). These analyses were conducted to control for this difference, and thus to ensure that any prediction effects arose from differences between what was expected and what was shown, rather than by the degree of difference between previous and current stimulus locations.

Specifically, this analysis considered the representation of stimulus location to be dependent on the location of the previous stimulus in the sequence. Thus, we considered eight different location conditions: each of the four distinct experimental positions preceded by the stimulus 90° clockwise and 90° counterclockwise, which corresponded with the expected stimulus locations within the Testing Block (corresponding with the eight arrows in Fig. 1C). Classifiers were trained to distinguish these adjacent stimulus pairs (previous/current) using epochs in which these combinations occurred unpredictably (i.e., in the Random Block). These classifiers were then tested on Testing Block epochs in which these previous/current stimulus pairs were expected or unexpected.

**Fig. 5.**
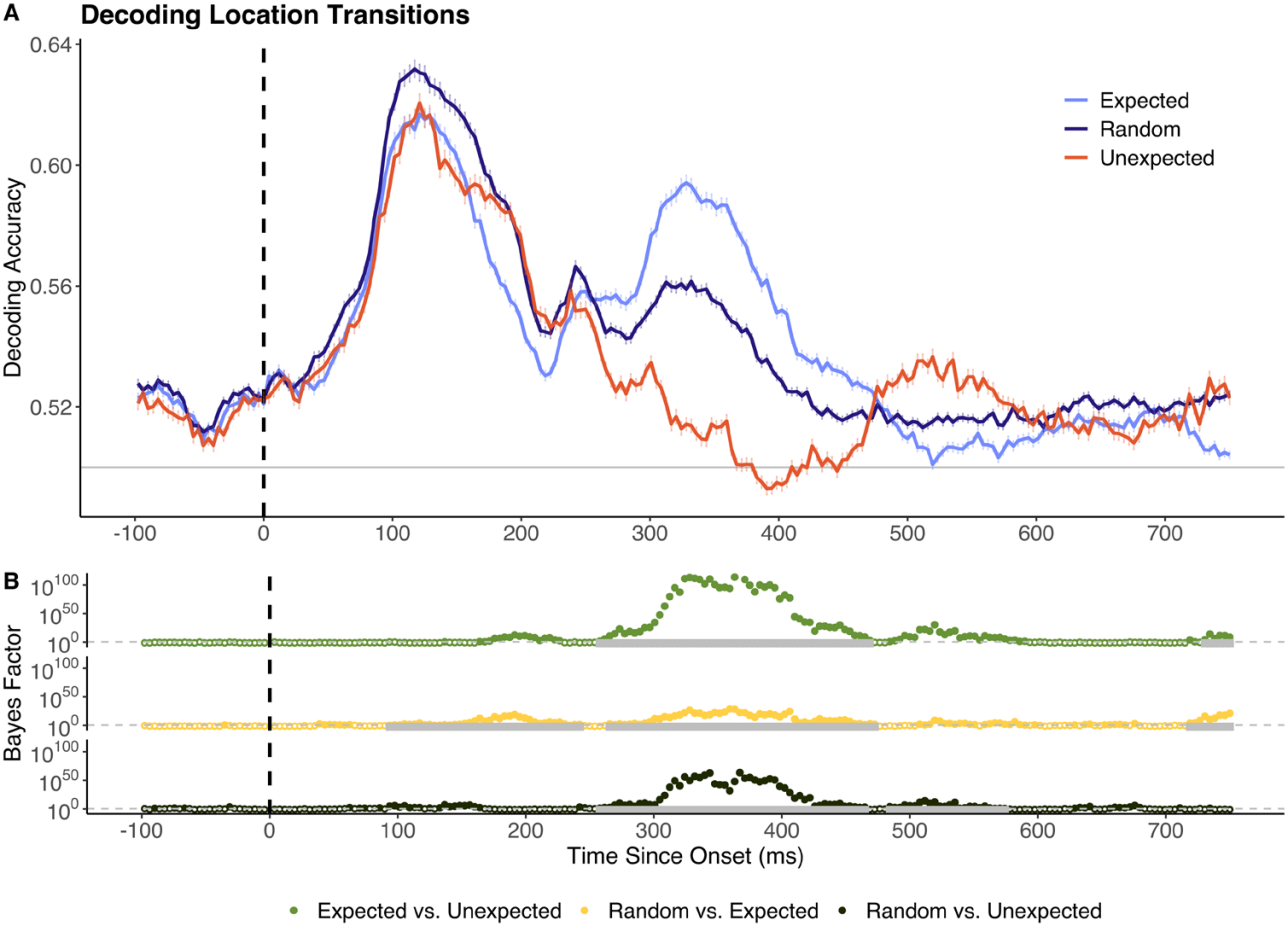
Decoding accuracy for random, expected, and unexpected location locations, controlling for the location of the previous stimulus. (A) Sample mean decoding accuracy across time (x-axis in ms). Chance performance (50%) is denoted in grey and stimulus onset is denoted by the dotted vertical line. (B) Bayes factor plots for between-model comparisons. The dotted grey line denotes the boundary for moderate evidence (BF = 3). Tests yielding BF > 3 are represented by filled dots. Time windows in which differences remained significant following cluster-based permutation corrections are highlighted in grey. All reported time points are rounded to the nearest millisecond.

As shown in Figure 5, when the location of the previous stimulus was controlled for, decoding accuracy for random locations was above chance in the pre-stimulus interval and throughout the entire epoch duration. This early above-chance decoding was expected because the analysis only included previous/current stimulus pairs that were 90° apart. This means that the stimulus displayed at -200 ms necessarily constrained the stimulus displayed at 0 ms (e.g., 90° could not be followed by 270°), thereby providing some predictive information about location before 0 ms. To control for this, decoding accuracy differences were evaluated relative to decoding accuracy for Random Block stimuli, rather than relative to chance. Decoding accuracy for expected stimuli was significantly lower than random between 93 and 242 ms after onset, and significantly higher than random between 265 and 472 ms after onset. Decoding accuracy was higher for unexpected relative to random stimuli within a similar later time window (257–464 ms). Decoding performance for expected and unexpected stimuli was significantly different between 257 and 468 ms after onset, with expected stimuli yielding higher decoding accuracies.

### 3.4 Investigating the representational time course of prediction effects

Time generalisation analyses were conducted to evaluate whether prediction condition modulated the representational time course of stimulus location information (Carlson et al., 2020; King & Dehaene, 2014). In this analysis, decoding accuracy was computed for all possible combinations of time points. Specifically, classifiers were trained to distinguish locations based on data collected at each time point independently, and each of these models was tested on data available at every other time point. In cases where little temporal generalisation is present, peak decoding accuracy is expected to align with the diagonal (where classifiers are trained and tested at the same time points).

**Fig. 6.**
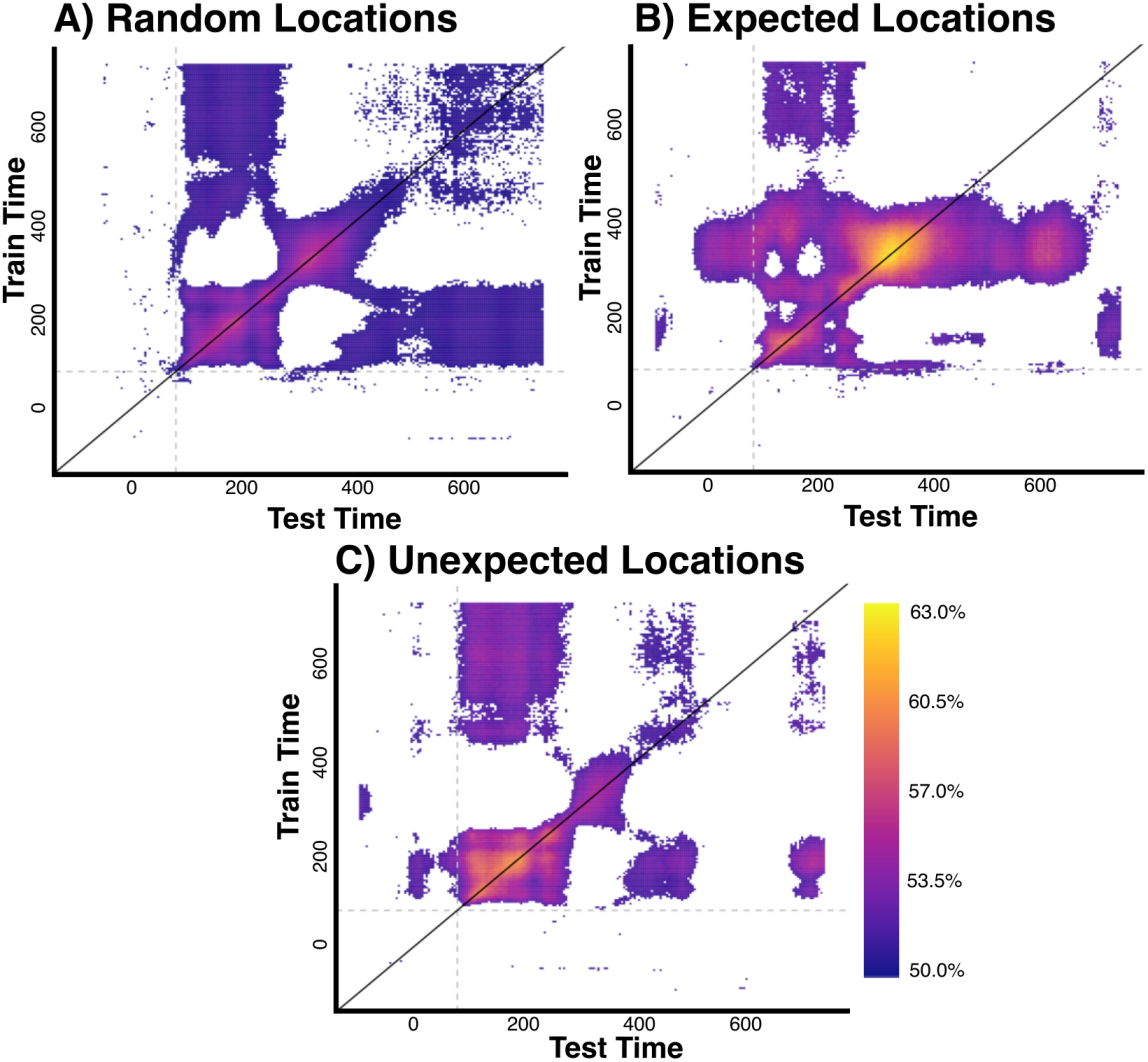
Time generalisation plots for location decoding across different prediction conditions. Decoding accuracy for models trained at specific times (y-axis) and tested at different time points (x-axis). Dotted grey lines denote the expected minimum onset time of above-chance decoding accuracy (80 ms, Carlson et al., 2013). Pixel colour denotes mean decoding accuracy at each time point. Only models yielding above-chance decoding accuracy (mean > 50% and BF > 10) are shown here. (A) Results for random locations, suggesting limited model generalisation across time. (B) Results for expected locations, suggesting that models trained on data presented between 250 and 400 ms generalised to times before the expected window for representation (<80 ms) and later times (>400 ms). (C) Results for unexpected locations, demonstrating limited generalisation across time.

Time generalisation analyses for random and unexpected location events yielded above-chance decoding accuracies that were primarily centred around the diagonal, implying little generalisation between models trained and tested on early versus later time points (Fig. 6A & C). Time generalisation analyses of expected locations (Fig. 6B) yielded above-chance decoding accuracy centred along the diagonal, in addition to both early (<80 ms) and later (>400 ms) generalisation of models trained on time points between 250 and 400 ms following onset.

Finally, exploratory searchlight decoding analyses were conducted to investigate which features (i.e., EEG channels) most strongly influenced classification and whether these topographies varied across stimuli in different expectation conditions. Differences in decoding accuracy could conceivably be driven by changes in the fidelity of represented information, or by changes in the spatial topography of the represented information (Carlson et al., 2020). Classifiers trained on random location data might not effectively capture a change in spatial topography, since accuracy in the tested model will depend upon the actual channels carrying the most information in the random model. Searchlight analyses were, therefore, employed to investigate whether apparent decreases in decoding accuracy were due to changes in information quality or changes in the spatial distribution of information across the head. These analyses used classifiers trained on stimuli appearing in expected and unexpected sequence positions (rather than random data) to enable detection of spatial differences in information topographies between these conditions. Local clusters were created by sequentially selecting each EEG channel and its closest four neighbouring channels. Classifiers were trained and tested to distinguish between locations based on data from each cluster independently, resulting in a time-by-decoding accuracy channel map for each participant for stimuli in each prediction condition (expected, unexpected).

**Fig. 7.**
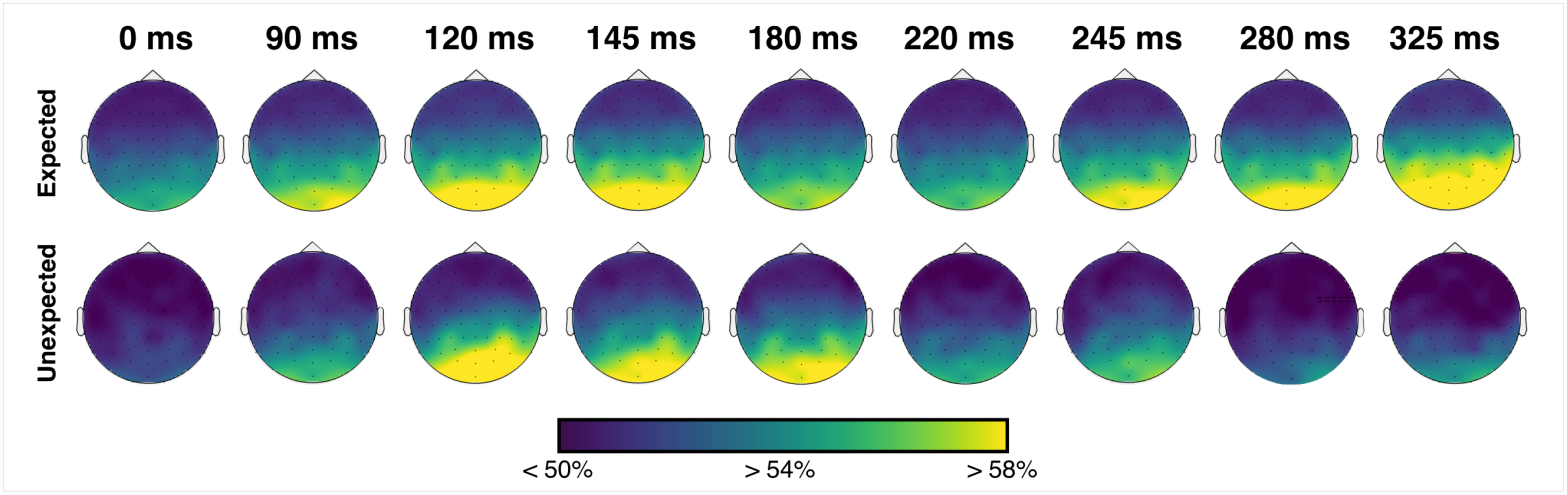
Decoding accuracy for searchlight classifiers trained to distinguish between different locations within expected and unexpected conditions independently. Colour denotes mean decoding accuracy at each defined local cluster at selected time points. Time points were selected to visualise key peaks and differences present in the decoding models visualised in Figure 2.

Classification accuracy was mainly driven by posterior (bilateral) electrodes across all prediction conditions and time points (Fig. 7). This result implies that key differences in decoding accuracy between expected and unexpected location stimuli were driven by differences in signal fidelity rather than differences in spatial topography.

## 4 Discussion

Our results reveal that predictive information dynamically modulates the representational time course of both “what” and “where” processing for familiar visual objects. Our approach enabled a detailed examination of two distinct sources of predictive information (object identity and location) within a single probabilistic cueing paradigm. The temporal decoding-based analyses provided a fine-grained characterisation of the representational time course of predictive processes, providing new insights into the processing stages at which these effects occur. Our results are important when considered in the context of predictive coding research, as they highlight complexities in how predictability modulates neural responses. The findings reveal that predictive information modulates representational fidelity when decoding both “what” and “where” visual information, but the occurrence, timing, and direction of these effects are distinct, as we describe below.

### 4.1 Prediction effects vary across different stimulus features

Decoding accuracy for object-level information was similar for expected versus random stimuli, but these values were significantly different in the location analyses. Some previous studies found limited or no evidence for decoding differences between expected and unexpected object stimuli (den Ouden et al., 2023, 2024). Recently, however, we found that expected object images had higher representational fidelity than random images, but only in cases where the images were warped to reduce recognisability (Moore et al., 2024). In this context, it is plausible that predictive information does not modulate the representational fidelity of expected objects relative to random objects when the stimuli are clearly discriminable.

However, it is also possible that the impact of predictive information on the representational fidelity of objects is comparatively small. Our object-level decoding classifiers were trained on fewer data epochs per location due to the need for object-level classifiers to be trained and tested on data from each spatial location independently (see Supplementary Table 1). These comparatively low trial numbers, in conjunction with reduced decoding accuracy due to off-fixation stimulus presentations, likely account for the comparatively variable decoding performance found in object-level analyses (Robinson et al., 2025; Whyte et al., 2020). While the classifiers reliably produced above-chance deducing accuracy, the overall low decoding performance implies that only comparatively large prediction effects would be detectable in our paradigm. In this context, it is plausible that prediction has a modulatory effect on object representations, but that the experimental design employed here was not sufficiently sensitive to fully capture this potentially small effect. Future work could develop similar paradigms with improved object-level decoding performance to evaluate this possibility. This could be achieved by maximising pair-wise decoding performance by selecting highly decodable object stimuli (e.g., faces versus houses) or by decoding centrally presented objects rather than objects in the visual periphery.

In contrast to the object-decoding findings, there were robust effects of expectancy in the location-level analyses, and these varied dynamically for several hundred milliseconds after stimulus onset. Specifically, location-decoding differences were consistent with expectation suppression in a short, early time window (160–238 ms), but this effect was not consistent across the majority of the time window yielding above-chance decoding. Notably, the identified early prediction effects (<150 ms) were not replicated when the location of previous stimuli was controlled for (Fig. 5). This suggests that, to some extent, early expectation suppression-like decoding differences may reflect the degree of spatial difference between previous and current stimuli, rather than indexing the differences between what was expected and what was presented.

At later time windows, however, representations of expected location stimuli were comparatively enhanced. This effect remained consistent when the location of previous stimuli was controlled for. Time generalisation analyses also indicated that that the location of expected stimuli is represented in neural activity present earlier than the expected onset of above-chance decoding accuracy, as found in previous work (Blom et al., 2020; Hogendoorn & Burkitt, 2018). Expectation suppression is a core hypothesis arising from the predictive coding framework (Millidge et al., 2021; Summerfield & Egner, 2009), and posits that expected stimuli elicit reduced neural responses relative to stimuli which appear randomly (i.e., are neither expected nor unexpected; Smout et al., 2019; Tang et al., 2018, 2023). Our findings for location information are broadly consistent with past work which has suggested that neural prediction effects do not always align with the hypothesised effects arising from expectation suppression (den Ouden et al., 2023, 2024; Moore et al., 2024). In contrast to predictive coding accounts, attention-based models of expectation suppression postulate that reduced responses to expected stimuli are due to reduced allocation of attentional resources to predictable events (Alink & Blank, 2021). This account suggests that unexpected stimuli should yield stronger decoding relative to expected stimuli, as they are allocated greater attentional resources. This idea is not supported by the current results, as we found that decoding accuracy for unexpected stimuli was generally lower than for expected and random stimuli.

Across stimulus identity and location analyses, there was consistent evidence for reduced decoding of unexpected relative to random stimuli in later time windows (>250 ms). This effect was more pronounced for location than for object information but remained statistically significant for both. Previous work investigating the representational dynamics of object stimuli similar to those used here reported that this later time window (>250 ms) may relate to higher-level categorical representations (Carlson et al., 2013). For example, peak decoding occurs at 240 ms post-onset when classifying whether images depict animate or inanimate objects (Carlson et al., 2013; Contini et al., 2021; Grootswagers et al., 2019). Decoding accuracy for other intermediate-level semantic categories (e.g., animals, humans) have also been found to peak at around this time (Carlson et al., 2013).

In this context, the later (>250 ms) reduced decoding for unexpected objects may correspond to changes in the representation of higher-level categorical information rather than lower-level visual features. In terms of object-level decoding results, this implies that object-level predictions may be operationalised as encoding of expectations about the higher-level semantic content of an image rather than a specific pattern of expected low-level features (Moore et al., 2024). However, it is unclear what specific processing elements a later (>250 ms) decoding difference might reflect in the context of location-level analyses in which high-level categorical information is not predictable. It is also plausible that later differences in signal fidelity reflect the representation and/or transmission of general prediction signals such as prediction error, model updating across the visual hierarchy, or attentional orienting differences (Moerel et al., 2022; Summerfield & Egner, 2009; Whyte et al., 2020). However, for location-decoding analyses, the representational time course of unexpected stimuli varied across different types of location violations (e.g. -90° vs +/-180° relative to the expected location; see Supplementary Fig. 2). This may have arisen because for cases in which a stimulus appeared at an unexpected location, the next stimulus in the sequence commenced again from a predictable location (clockwise or counterclockwise). For example, for an unexpected stimulus located at -90°, the stimulus preceding the violation could be repeated immediately after the violation (i.e., 90° at -200 ms, 0° at 0 ms (the unexpected stimulus), and 90° again at +200 ms). This pattern may have influenced decoding performance, as the neural response present from ~300 ms after onset of an unexpected stimulus might also have captured information that was systematically different from the representation of the unexpected stimulus alone.

The current findings also have important implications for future studies aimed at characterising prediction-related differences in visually evoked neural activity. For studies employing decoding methodologies, the specific contrast being decoded is critically important. Although prediction condition modulates the representational fidelity of stimulus-specific information, it does not seem to yield activity differences which can reliably distinguish all stimuli that are expected from all stimuli that are unexpected (Fig. 4). This finding is in line with the assertations of predictive coding theory, as the information carried by prediction error signals is expected to vary based on the degree of mismatch between what was expected and what was presented (Millidge et al., 2021; Rao & Ballard, 1999).

### 4.2 Prediction effects are temporally dynamic

It is possible that differences between early (<150 ms) and later (>150 ms) prediction effects documented in this study represent different neural aspects of predictive perception. Past work has suggested that prediction modulates two aspects of violation-related neural responses: task-related attention and stimulus-evoked response modulations (Feuerriegel et al., 2021; Press et al., 2020; Rungratsameetaweemana & Serences, 2019). In terms of task-related attention, unexpected stimuli may be relatively salient and, therefore, attract attention (Press et al., 2020; Richter & de Lange, 2019). Increased attention is associated with enhanced responses in stimulus-selective neurons, and higher decoding accuracy relative to unattended stimuli (Corbetta et al., 1990; Grootswagers et al., 2021; Smout et al., 2019). These task-related attention effects are thought to arise early in the representational time course, as soon as a discrepancy between expected and presented information is detected (Feuerriegel et al., 2021; Rungratsameetaweemana & Serences, 2019).

Conversely, stimulus-evoked neural modulations relate to changes in how information about the identity of presented stimuli is represented across different expectation conditions (Rungratsameetaweemana & Serences, 2019). In the context of predictive coding, these stimulus-evoked effects would be expected to involve both changes in the representational fidelity of stimulus information and the encoding of prediction error and other signals related to updating predictive models (Summerfield & Egner, 2009). Past work has suggested that these stimulus-evoked response modulations occur at a comparatively later time point as discrepancies between expectations and stimulus input must be detected before prediction errors can be integrated to update perceptual models (Feuerriegel et al., 2021; Rungratsameetaweemana & Serences, 2019).

Past work has indicated that prediction-related modulatory effects (e.g. expectation suppression) are dissociable from task-related attention or surprise effects (Kaposvari et al., 2018; Ramachandran et al., 2016). In this context, it is plausible that our finding of different prediction effects in early (>150 ms) and later (>150) time points is due to modulations of task-related attention and stimulus-specific representations, respectively (Moerel et al., 2022). However, this possibility remains speculative as the extent to which attention and stimulus-specific modulations can be temporally dissociated was not explicitly evaluated in this study. Future work could explicitly manipulate attention to obtain a better understanding of the temporal dynamics of prediction-related attention and whether this effect can be temporally dissociated from other neural responses related to prediction modulation.

Our experiment employed a probabilistic cueing design in which participants were briefly exposed to six stimulus pairings with different statistical likelihoods. It is unclear whether the predictive relationships learned by participants were explicit or implicit, though in previous work using a similar design we found that participants were unable to report the statistical relationships between stimuli (Moore et al., 2024). In this context, we note that previous studies of predictive coding have varied stimulus expectancy in many different ways (Dehaene et al., 2015; Feuerriegel et al., 2021; Kok et al., 2014; Rideaux et al., 2025; Tang et al., 2018, 2023). Future work should aim to explore whether the results identified in the current study generalise to other types of predictive relationships.

## Supplementary Material

Supplementary Material

## Data Availability

All processed data and code associated with this project are openly available on the Open Science Framework (https://osf.io/s5nux/). EEG data are available on request from the authors.
