# Supplementary material for: Expectation dynamically modulates the representational time course of objects and locations
Source: Imaging Neurosci (Camb). 2025 Nov 10;3:IMAG.a.999. doi: 10.1162/IMAG.a.999 (PMC12603655; doi:10.1162/IMAG.a.999)
Supplement: Supplementary Material [file IMAG.a.999_supp.pdf]

Supplementary Materials:

Training Data	Testing Data	Figure	N Epochs	Onset	End	Peak Accuracy	Peak Time	N BF > 3
Location-Level Analyses:								
Random Locations		2	2157.3 (1631 - 2435)	78		58.40%	324	237
Random Locations	Expected Locations	2	372.8 (187 - 452)	66	492	61.40%	328	193
Random Locations	Unexpected Locations	2	372.8 (187 - 452)	74	558	59.80%	191	185
Expected vs. Unexpected Locations		4				51.00%	188	6
Random (Controlled)		5	1080.4 (771 - 1241)	-100		63.20%	117	282
Random (Controlled)	Expected (Controlled)	5	185.2 (89 - 245)	-100		61.70%	121	272
Random (Controlled)	Unexpected (Controlled)	5	185.2 (89 - 245)	-100		62.00%	121	260
Object-Level Analyses:								
Random Objects		3	266.5 (188 - 320)	101	445	52.90%	246	99
Random Objects	Expected Objects	3	150.4 (70 - 218)	93	449	52.20%	238	99
Random Objects	Unexpected Objects	3	150.4 (70 - 218)	97	433	52.10%	254	102

Supplementary Table 1: *Summary of the 10 reported decoding analyses. 'Training Data' corresponds to the stimulus types used to train the classifier, whereas 'Testing Data' refer to the stimulus types used to test the model. Where testing data are not reported, models were trained and tested on the same stimuli. 'N Epochs' refers to the number of epochs (per participant) used to train or test each classifier. 'Onset' indicates the first time at which at least three sequential timepoints were significantly above chance. Onsets are not reported for analyses in which no timepoints survived corrections for multiple comparisons. 'End' refers to the last timepoint at which at least three sequential timepoints were above chance. In cases where 'End' is not reported, decoding accuracy remained above chance until the end of the relevant epoch. 'Peak Accuracy' is the maximum mean classifier accuracy, and 'Peak Time' is the time at which this occurred. 'N BF > 3' reports the number of timepoints at which decoding accuracy was above chance (BF > 3) between -100 and 1000ms from stimulus onset. 'Controlled' analyses denote cases in which analyses controlled for the location of the previous stimulus.*

Prediction effects for best and worst performers in the attention probe task

Exploratory analyses were conducted to assess whether prediction effects differed for high- and low-performers on the attention probe task, with the aim of identifying any modulatory influence of task-related attention.

First, a regression analysis was conducted to assess whether the degree of expectation suppression was correlated with task performance. The magnitude of expectation suppression was quantified by calculating the mean accuracy difference between random and expected

location stimuli at each timepoint within the time window where expectation suppression was present at the group level (160 – 238ms; see Figure 2). There was no association between the degree of expectation suppression and accuracy in the attention probe task ($F(1,38) = 0.475$, $p = 0.495$. adjusted R-squared = -0.01).

Next, the location-level decoding analyses (see Figure 2) were repeated with data from participants in the top quartile (accuracy > 91.0% , n = 7) and bottom quartile (accuracy < 74.6%, n = 7) of the group, as assessed by the attention probe task. Temporally dynamic decoding effects similar to those identified in the entire sample were evident in both the best and worst performers (Supplementary Figure 1). Specifically, for both subgroups, decoding accuracy for expected stimuli was lower than for random stimuli in an early time window (<250 ms), and higher relative to random in a later time window (> 250ms). Decoding accuracy differences between random and unexpected stimuli were larger for the best performers relative to the worst performers. Notably, for the best performers, decoding accuracy for unexpected stimuli was briefly below chance before 100ms, but this effect was not apparent for the worst performers.

We recommend caution in interpreting the results of these additional exploratory analyses, for two reasons. First, the attention task we employed required participants to count the number of probes (red stars) over lengthy (~45 second) trial sequences, so it cannot be used to infer moment-to-moment fluctuations in attentional state. Second, our strategy of separating participants into best and worst performers necessarily yielded very small sample sizes, with a consequent increase in variability.

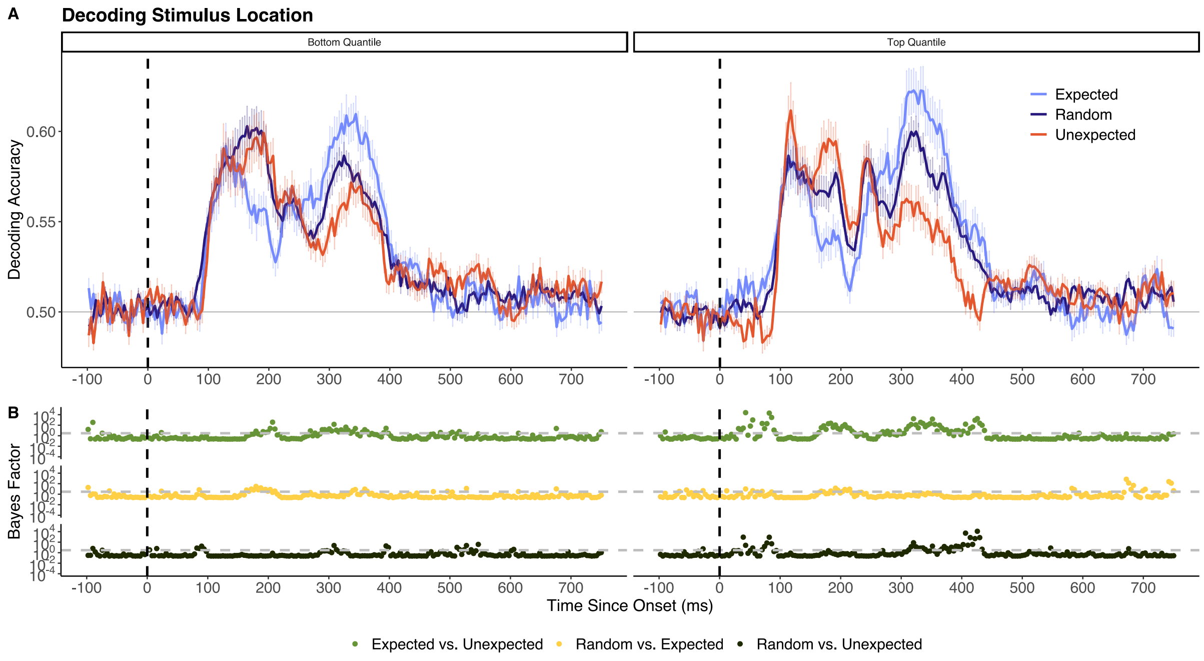

Supplementary Figure 1: *Decoding accuracy for stimuli at random, expected, and unexpected locations for the best and worst performing participants in the attention probe task (top versus bottom quartile; n=7 in each group). (A) Sample mean decoding accuracy across time (x-axis in ms). Chance performance (50%) is denoted in grey, and stimulus onset is denoted by the dotted vertical line. (B) Bayes factor plots for between-model comparisons. The dotted grey line denotes the boundary for moderate evidence (BF = 3). All reported timepoints are rounded to the nearest millisecond.*

Prediction effects differ between different types of unexpected location stimuli:

Unexpected location violations could either involve transitions of -90 degrees or +/-180 degrees relative to the previously displayed stimulus. These different transitions could plausibly have been associated with different representational time courses due to the location-level predictive structure used in the experiment. This may have arisen because for cases in which a stimulus appeared at an unexpected location, the next stimulus in the sequence commenced again from a predictable location (clockwise or counterclockwise). For example, for an unexpected stimulus located at -90°, the stimulus preceding the violation could be repeated immediately after the violation (i.e., 90° at -200 ms, 0° at 0ms (the unexpected stimulus), and 90° again at +200ms). This pattern may have influenced decoding performance within -90° unexpected stimulus, as the neural response present from ~300ms after onset of an

unexpected stimulus might also have captured information that was systematically different from the representation of the unexpected stimulus alone.

To investigate this possibility, the decoding analyses for unexpected and random stimuli reported in Figure 2 were repeated for stimulus transitions at -90° and +/-180° separately. In these analyses, classifiers were trained to distinguish stimuli for which transitions occurred randomly (i.e., stimuli from the Random Block; see Methods) and were tested on unexpected stimuli from the Testing Block. Decoding accuracy for random vs unexpected stimuli differed across these two possible transition types (see Supplementary Figure 2). For -90° transitions, decoding accuracy for unexpected stimuli was lower than for random stimuli between 269 and 425 ms post-onset. By contrast, for +/-180° transitions, decoding accuracy for unexpected stimuli was lower than for random stimuli between 101 and 203 ms post-onset.

The later (>250ms) reduction in decoding accuracy for unexpected relative to random stimuli observed at the group level was only present for -90° violations in these analyses. Importantly, for the -90° violation, the previously presented (-200ms) stimulus was repeated at 200ms. Thus, the decrease in decoding accuracy after this time might have arisen at least in part because of this repeated stimulus. Taken together, these supplementary findings imply that at least a portion of the group-level decoding for unexpected stimuli might have arisen from these -90° violations.

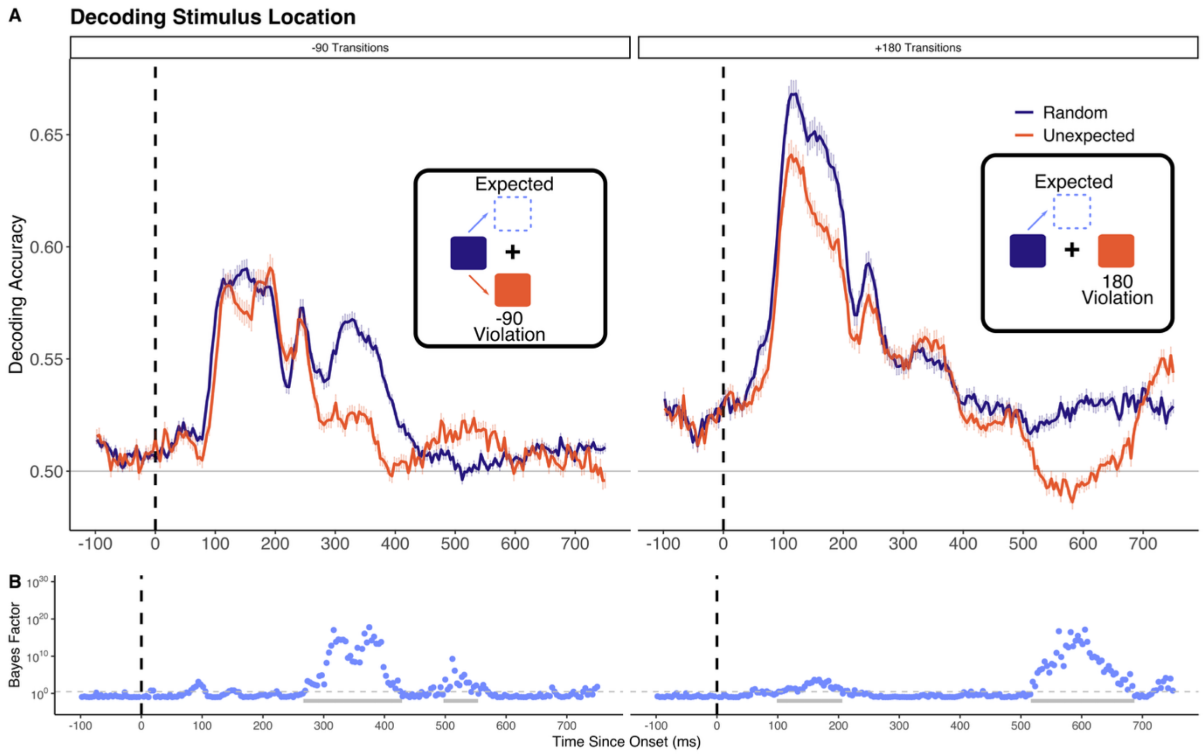

Supplementary Figure 2: *Decoding accuracy for random, expected, and unexpected locations for -90° and +/-180° expectation violations. (A) Mean decoding accuracy across time (x-axis in ms). Chance performance (50%) is denoted in grey and stimulus onset is denoted by the dotted vertical line. (B) Bayes factor plots for between-model comparisons. The dotted grey line denotes the boundary for moderate evidence (BF = 3). Time windows in which differences remained significant following cluster-based permutation corrections are highlighted in grey. All reported timepoints are rounded to the nearest millisecond.*
